# Vitamin D deficiency leads to the abnormal activation of the complement system

**DOI:** 10.1007/s12026-022-09324-6

**Published:** 2022-09-30

**Authors:** Huan Li, Xiaomin Xie, Guirong Bai, Dan Qiang, Li Zhang, Huili Liu, Yanting He, Yanpan Tang, Ling Li

**Affiliations:** Department of Endocrinology, The First People’s Hospital of Yinchuan, Ningxia Hui Autonomous Region, No. 2, Liqun West Street, Xingqing District, Yinchuan, 750001 China

**Keywords:** Vitamin D, Insulin resistance, Glucose metabolism, Complement system, Immune system

## Abstract

Vitamin D deficiency can damage the human immune system, and the complement system is a key component of the immune system. This study aimed to elucidate the mechanism by which vitamin D affects the immune system by analyzing the changes in the protein expression of the complement system under different vitamin D levels. We selected 40 participants and divided them into three groups according to their serum levels of 25-hydroxyvitamin D (25(OH)VD): group A, 25(OH)VD ≥ 40 ng/mL; group B, 30 ng/mL ≤ 25(OH)VD < 40 ng/mL; and group C, 25(OH)VD < 30 ng/mL. Serum samples were subjected to biochemical analysis, followed by proteomic analysis using high-throughput untargeted proteomic techniques. Vitamin D deficiency increased the levels of fasting blood sugar, fasting serum insulin, and homeostasis model assessment (HOMA) of insulin resistance and decreased the secretion of HOMA of β-cell function, which led to insulin resistance and glucose metabolism disorder. Moreover, vitamin D deficiency resulted in the abnormal expression of 56 differential proteins, among which the expression levels of complement factor B, complement component C9, inducible co-stimulator ligand, and peptidase inhibitor 16 significantly changed with the decrease in vitamin D content. Functional enrichment analysis of these differential proteins showed that they were mainly concentrated in functions and pathways related to insulin secretion and inflammation. In conclusion, vitamin D deficiency not only contributes to insulin resistance and glucose metabolism disorder but also causes abnormal protein expression, resulting in the abnormal activation of the complement system. This study provides a novel theoretical basis for further studies on the relationship between vitamin D and the immune system.

**Supplementary Information:**

The online version contains supplementary material available at 10.1007/s12026-022-09324-6.

## Introduction

Vitamin D is essential for maintaining human health [1]. Early evidence suggests that vitamin D plays an important role in promoting innate immunity [2]. As a regulator of innate immunity, vitamin D can promote the production of defensin β2 and cathelicidin antimicrobial peptides by macrophages and monocyte keratinocytes to increase antimicrobial activity [3, 4]. In addition, vitamin D regulates the innate immune system and increases the phagocytic ability of immune cells [5, 6]. It also suppresses the adaptive immune system by inhibiting T helper type 1 (Th1)/Th17 cells and promoting the growth of regulatory T cells (Tregs) [7]. Currently, the main sources of vitamin D are natural foods, endogenous production that relies on ultraviolet B, and dietary supplements [8]. However, vitamin D deficiency remains a major public health problem worldwide. Generally, vitamin D deficiency is associated with various autoimmune diseases [9]. For instance, vitamin D deficiency is common in patients with active ulcerative colitis [10]. Vitamin D deficiency in pregnant women may influence the spectrum of Tregs [11], which can cause impaired immunosuppressant functions [9]. Understanding the mechanism by which vitamin D deficiency affects the immune system is important.

The complement system, a component of innate immunity, is a bridge between innate and adaptive immunity, providing the first line of defense against microorganisms, and is a necessary process to clear apoptotic cells and immune complexes [12]. In a healthy state (i.e., in the absence of a trigger), the complement system maintains a resting state through a series of liquid phase and cell surface regulatory proteins [13, 14]. However, the complement system is activated immediately when the immune system is attacked, with the production of complement cutting fragments and depletion of complement components [15, 16]. Various autoimmune diseases, such as systemic lupus erythematosus and paroxysmal nocturnal hemoglobinuria, are associated with the abnormal activation of the complement system [17, 18]. Patients with diabetic neuropathy also exhibit an abnormal activation of the complement system [19]. Vitamin D and the complement system reportedly share some common pathophysiological pathways in the musculoskeletal system, circulation, and metabolism [20]. Small et al. demonstrated that vitamin D could upregulate the innate immunity of macrophage complement receptor immunoglobulin to pathogenic microorganisms [21]. However, whether vitamin D deficiency causes the abnormal activation of the complement system is unclear.

In the present study, we applied high-throughput untargeted proteomic technology to group a physical examination population under different fasting glucose states without any intervention according to their vitamin D levels and to explore the possible mechanism of vitamin D deficiency on the activation of the human complement system.

## Materials and methods

### Research subjects

Individuals (22 males and 18 females aged 27–57 years) of a physical examination population from Yinchuan First People’s Hospital, the Second Affiliated Hospital of Ningxia Medical University, were enrolled in this study. Among them, 10 had fasting blood sugar (FBG) < 5.6 mmol/L, 10 had 5.6 mmol/L ≤ FBG < 6.1 mmol/L, 10 had 6.1 mmol/L ≤ FBG < 7.0 mmol/L, and 10 had FBG ≥ 7.0 mmol/L. The diagnostic criteria of type 2 diabetes and prediabetes met the diagnostic criteria of the American Diabetes Association in 2019 [22]. All participants were divided into three groups according to their serum levels of 25-hydroxyvitamin D (25(OH)VD): group A (*n* = 15), 25(OH)VD ≥ 40 ng/mL; group B (*n* = 15), 30 ng/mL ≤ 25(OH)VD < 40 ng/mL; and group C (*n* = 10), 25(OH)VD < 30 ng/mL. All participants did not receive any lifestyle intervention and drug treatment. Patients with the following characteristics were excluded: previously diagnosed prediabetes and diabetes; kidney, liver disease, or cancer; acute or chronic infection or acute or chronic inflammatory disease; a history of cardiovascular and cerebrovascular diseases; thyroid dysfunction; any blood disease; alcohol or drug abuse or smoking; and hormone replacement therapy. In addition, patients who had been supplemented with vitamin D in the recent 3 months were excluded. This project was carried out in accordance with the Provisions of the Declaration of Helsinki and was approved by the Ethics Committee of Yinchuan First People’s Hospital. All participants provided informed consent. All study groups were matched in terms of gender and age, and all parameters showed no statistically significant differences.

### Collecting data

All participants were asked to answer a unified questionnaire to collect general data regarding their gender, age, smoking history, systolic blood pressure (SBP), diastolic blood pressure (DBP), height, weight, waist circumference and hip circumference, body mass index (BMI), and waist hip ratio (WHR). Peripheral venous blood was collected from all participants after fasting for 8–12 h to detect fasting blood glucose (FBG), total cholesterol (TC), low-density lipoprotein (LDL), high-density lipoprotein (HDL), triglyceride (TG), and urea (Beckman coulter AU5800). Serum samples from all participants were stored in a refrigerator at − 80 °C for subsequent analyses.

### Enzyme-linked immunosorbent assay (ELISA)

The concentrations of fasting serum insulin (FINS), glycosylated hemoglobin (HbA1c), 25-hydroxyvitamin D (25(OH)-VD), alanine aminotransferase (ALT), and aspartate aminotransferase (AST) were measured using ELISA kits (eBioscience) in accordance with the manufacturer’s instructions.

### Islet beta-cell function

Homeostasis model assessment (HOMA) was used to assess insulin resistance and secretion from the fasting glucose and insulin concentrations using the following formulas: HOMA of insulin resistance (HOMA-IR) = FBG × FINS/22.5 and HOMA of β-cell function (HOMA-β) = 20 × FINS/(FPG − 3.5). The basic information of all of the above samples is listed in Table S1.

### Protein sample preparation

The samples were added to the high-abundance kit, incubated at 25 °C for 10 min, and then centrifuged at 1000 g for 2 min. The supernatant was obtained. Protein concentration was measured using the Bradford method [23]. In short, the samples were first diluted with lysis buffer, and then, the samples and standard were diluted with protein quantitative dye to avoid light reaction for 10 min. Absorbance was obtained at 595 nm using an enzyme-labeling instrument in accordance with the standard curve to calculate the sample concentration. Bovine serum albumin served as the standard. Subsequently, 2 μg of each sample was obtained for sodium dodecyl sulfate–polyacrylamide gel electrophoresis.

The extract of each sample was reduced by dithiothreitol at 60 °C for 1 h. Then, iodoacetamide was added to alkylated cysteine, and their mixture was incubated in a dark room at 20 °C for 1 h. Protein was diluted with NH_4_HCO_3_ and digested with trypsin at 37 °C for 16 h according to the protein/trypsin 50 (w/w) ratio. The polypeptide was acidified with formic acid (10%, v/v), desalted by reverse phase extraction with the tip of a C18 ZipTip pipette, and then resuspended in 0.1% FA for high-performance liquid chromatography tandem mass spectrometry (HPLC–MS/MS).

### Analysis of HPLC–MS/MS

An HPLC–MS/MS system coupled with a Q Exactive mass spectrometer was employed for label-free analysis (Thermo Scientific). The samples were injected by an automatic injector through programmed injection and then preconcentrated on a self-made C_18_ trap column. Then, they were analyzed on a self-made analytical column, in which the mobile phases were solvents A and B. The isolated peptide fragments were identified using Q Exactive HF MS/MS. Sequencing was performed using BMKcloud (Beijing China).

### Data reorganization

Protein identification and label-free quantification were performed using Proteome Discoverer (v2.1.0.81). A similarity search was performed against the forward UniProt database for *Homo sapiens* (UP000005640). The differentially expressed proteins between the A, B, and C group with |fold change > 1.2 and *p*-value < 0.05 as the inclusion criteria. The mass spectrometry proteomics data have been deposited to the ProteomeXchange Consortium via the PRIDE partner repository with the dataset identifier PXD036152.

### Functional enrichment analysis of differentially expressed proteins

The functional characteristics of differentially expressed proteins were comprehensively understood through the annotations of Gene Ontology (GO) protein, protein homologous group, Kyoto Gene Genome Encyclopedia (KEGG), and InterPro. Differentially expressed proteins were classified into biological process (BP), cell composition (CC), and molecular function (MF) through GO functional enrichment analysis. The signaling pathways related to differentially expressed proteins induced by vitamin D deficiency were discussed through KEGG enrichment analysis.

### Statistical analysis

Statistical analysis was conducted using SPSS 26.0 (IBM, USA) software, and all data were expressed as mean ± standard deviation. Non-normal distribution data were transformed into normal distribution by logarithm. In addition, principal component analysis (PCA) and cluster analysis were conducted using meta-analysis 3.0 online software. Different groups were compared using analysis of variance. Chi-square test was used to compare rates. Statistical significance was considered at *p* < 0.05.

## Results

### Vitamin D deficiency causes insulin resistance

The general information of the three groups of human samples is shown in Table 1. We compared the general data of the three groups and found no significant differences in age, SBP, DBP, BMI, WHR, TC, TG, HDL, LDL, ALT, AST, and UREA among the groups (*p* > 0.05). The levels of HbA1c, FBG, FINS, and HOMA-IR increased with decreasing vitamin D content, whereas that of HOMA-β showed the opposite (*p* < 0.05). These results suggest that vitamin D deficiency contributed to elevated blood sugar levels and insulin resistance.

**Table 1:** Clinical information of patients

	A (*n* = 15)	B (*n* = 15)	C (*n* = 10)	*F*	*p*
25(OH)VD (ng/mL)	49.12 ± 6.64	36.10 ± 3.72	22.21 ± 3.83	86.09	< 0.01
Age (years)	44.93 ± 8.67	43.47 ± 6.66	44.90 ± 8.72	0.155	0.857
SBP (mmHg)	118.40 ± 7.42	118.40 ± 10.11	121.90 ± 10.71	0.526	0.596
DBP (mmHg)	75.00 ± 9.35	73.07 ± 8.15	75.00 ± 6.13	0.260	0.772
BMI (kg/m^2^)	23.90 ± 3.60	23.42 ± 2.30	25.61 ± 3.75	1.455	0.246
WHR (cm/cm)	0.86 ± 0.07	0.82 ± 0.05	0.88 ± 0.03	3.001	0.062
TG (mmol/L)	2.01 ± 1.19	2.77 ± 3.98	2.29 ± 0.97	0.173	0.842
TC (mmol/L)	4.92 ± 1.07	5.35 ± 2.42	5.60 ± 1.29	0.490	0.617
HDL (mmol/L)	1.36 ± 0.30	1.33 ± 0.27	1.29 ± 0.17	0.233	0.793
LDL (mmol/L)	2.71 ± 0.78	3.33 ± 1.73	3.33 ± 0.96	1.224	0.306
ALT (µmol/L)	23.10 ± 7.13	23.55 ± 6.44	25.78 ± 4.53	0.724	0.501
AST (µmol/L)	25.57 ± 6.04	23.55 ± 6.44	25.78 ± 4.53	0.606	0.551
UREA (mmol/L)	4.68 ± 0.99	4.50 ± 1.16	4.81 ± 1.15	0.252	0.779
HbA1c (ng/mL)	172.17 ± 31.88	210.56 ± 30.34	247.04 ± 19.76	20.721	< 0.01
FBG (mmol/L)	5.44 ± 0.66	7.00 ± 2.98	10.46 ± 3.10	16.477	< 0.01
FINS (mIU/L)	4.99 ± 0.69	5.75 ± 0.71	6.43 ± 0.62	13.660	< 0.01
HOMA-IR	1.22 ± 0.30	1.84 ± 1.00	3.04 ± 1.08	18.212	< 0.01
HOMA-β	56.62 ± 18.83	44.48 ± 18.90	22.97 ± 11.67	14.912	< 0.01

### Vitamin D deficiency generates abnormal glucose metabolism

To further understand the effect of vitamin D deficiency on glucose metabolism, we subdivided each group of samples according to FBG values for subgroup analysis. As shown in Table 2, in group A, eight samples had FBG < 5.6 mmol/L and seven samples had 5.6 mmol/L < FBG ≤ 7.0 mmol/L. In group B, 2 samples had FBG < 5.6 mmol/L, 10 samples had 5.6 mmol/L < FBG ≤ 7.0 mmol/L, and 3 samples had FBG ≥ 7.0 mmol/L. In group C, three samples had 5.6 mmol/L < FBG ≤ 7.0 mmol/L and seven samples had FBG ≥ 7.0 mmol/L. The above results showed that vitamin D deficiency can lead to hyperglycemia and abnormal glucose metabolism.

**Table 2:** The proportion of different glucose metabolisms in each groups

	A (*n* = 15)	B (*n* = 15)	C (*n* = 10)	${\chi }^{2}$	***p***
FBG < 5.6 mmol/L	8	2	0		
5.6 mmol/L ≤ FBG < 7.0 mmol/L	7	10	3	21.80	< 0.001
FBG ≥ 7.0 mmol/L	0	3	7		

### Differential proteomic analysis

**Fig. 1:**
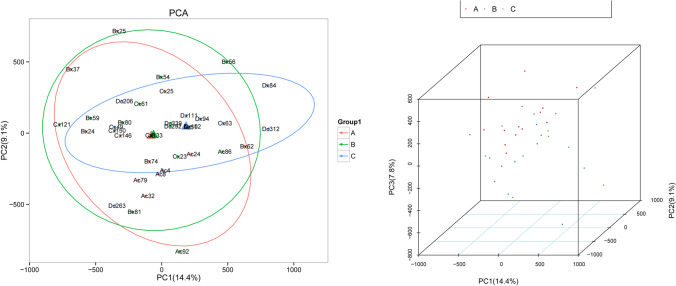
Principal component analysis (PCA) of the protein samples. Left, 2D plot of PCA; right, 3D plot of PCA

**Fig. 2:**
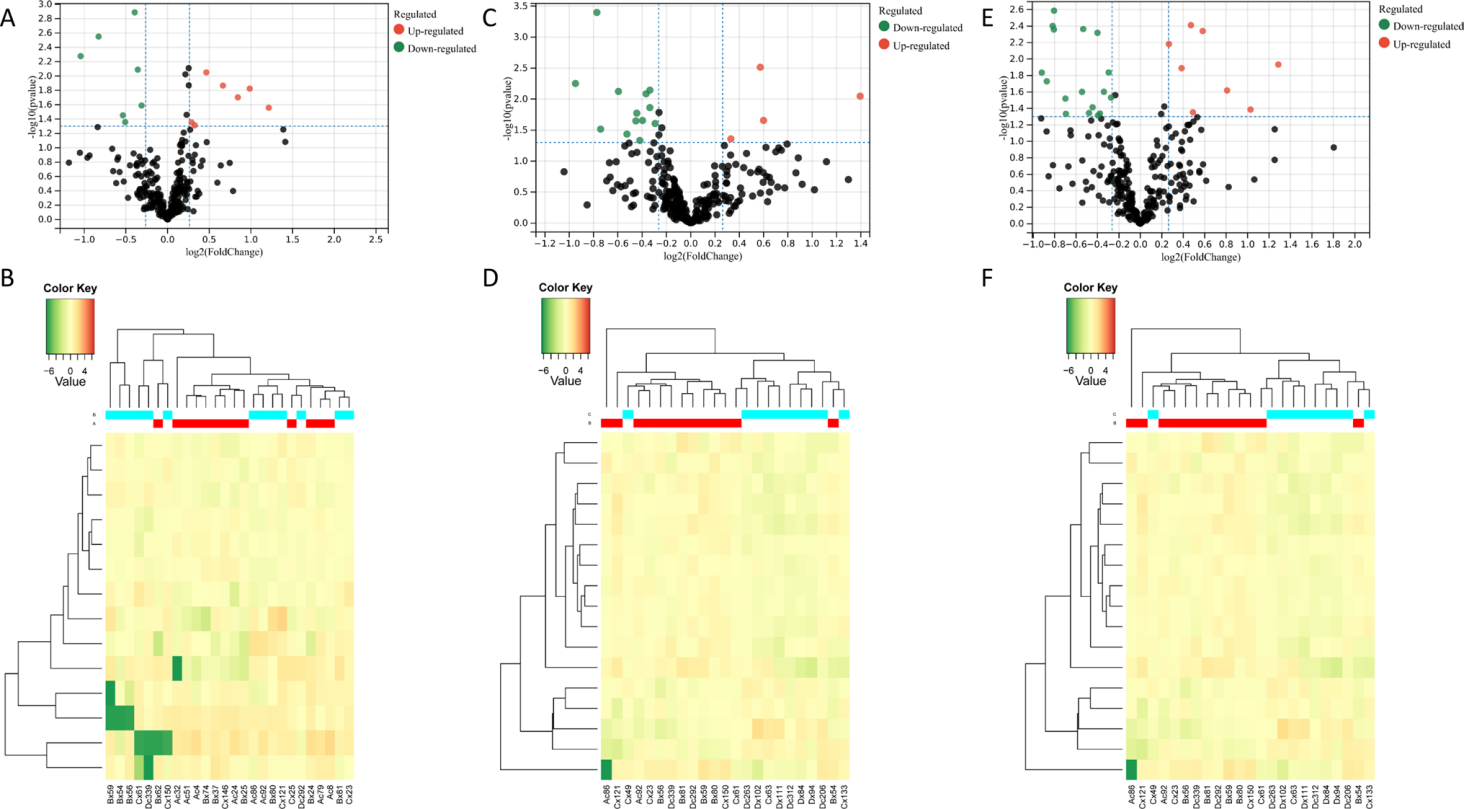
Analysis of the differentially expression proteins. **A** Volcano plot of differential proteins in A vs. B. **B** Cluster heatmaps of differential proteins in A vs. B. **C** Volcano plot of differential proteins in B vs. C. **D** Cluster heatmaps of differential proteins in B vs. C. **E** Volcano plot of differential proteins in A vs. C. **F** Cluster heatmaps of differential proteins in A vs. C. The green dot represents downregulated, and the red dot represents upregulated

After eliminating four outlier samples, PCA analysis was performed on the three groups of protein samples (Fig. 1). Proteomic differential analysis was performed to analyze the differential complement proteins affected by vitamin deficiency. As shown in the volcano plot (A vs. B), the expression levels of seven proteins were upregulated and the expression levels of seven proteins were downregulated with decreasing vitamin D content (Fig. 2A), such as complement factor B (CFB), tropomyosin alpha-4 chain (TPM4), and inducible co-stimulator ligand (ICOSL). Groups A and B were analyzed by heatmap overview, showing a good separation between the two groups (Fig. 2B). Similarly, compared with B group, 4 upregulated proteins and 13 downregulated proteins were found in group C (Fig. 2C), such as neural cell adhesion molecule L1-like protein and insulin-like growth factor I/II (IGF-I and IGF-II). Furthermore, more differential proteins were identified between groups A and C, and vitamin D deficiency upregulated the expression of 8 proteins and downregulated the expression of 17 proteins (Fig. 2E, A vs. C), such as CFB, immunoglobulin heavy constant alpha 2, complement component C9, peptidase inhibitor 16 (PI16), and ICOSL. The heatmap showed a good separation between groups B and C (Fig. 2D) and groups A and C (Fig. 2F). In addition, key proteins in the complement system were statistically analyzed (Table 3). To explore the effect of vitamin D on complement proteins, we listed the different complement proteins (Table 3). The results showed that vitamin D deficiency led to the abnormal expression of CFB, TPM4, C9, ICOSL, and PI16. These results suggest that vitamin D deficiency causes the abnormal expression of some proteins.

**Table 3:** Statistical table of differential complement proteins

Diff set	A vs. B	B vs. C	A vs. C
Complement factor B	Up (*p* = 0.04)	NS (*p* = 0.44)	Up (*p* = 0.01)
Coagulation factor XI	Up (*p* = 0.01)	NS (*p* = 0.46)	NS (*p* = 0.10)
Tropomyosin alpha-4 chain	Up (*p* = 0.01)	NS (*p* = 0.58)	Up (*p* = 0.02)
Inducible costimulator ligand	Down (*p* = 0.04)	NS (*p* = 0.70)	Down (*p* = 0.03)
Retinoic acid receptor responder protein 2	Down (*p* = 0.00)	NS (*p* = 0.07)	NS (*p* = 0.64)
Complement component C9	NS (*p* = 0.06)	NS (*p* = 0.17)	Up (*p* = 0.00)
Peptidase inhibitor 16	NS (*p* = 0.18)	NS (*p* = 0.37)	Down (*p* = 0.00)

*NS*, nonsignificant

### GO analysis of differentially expressed proteins

**Fig. 3:**
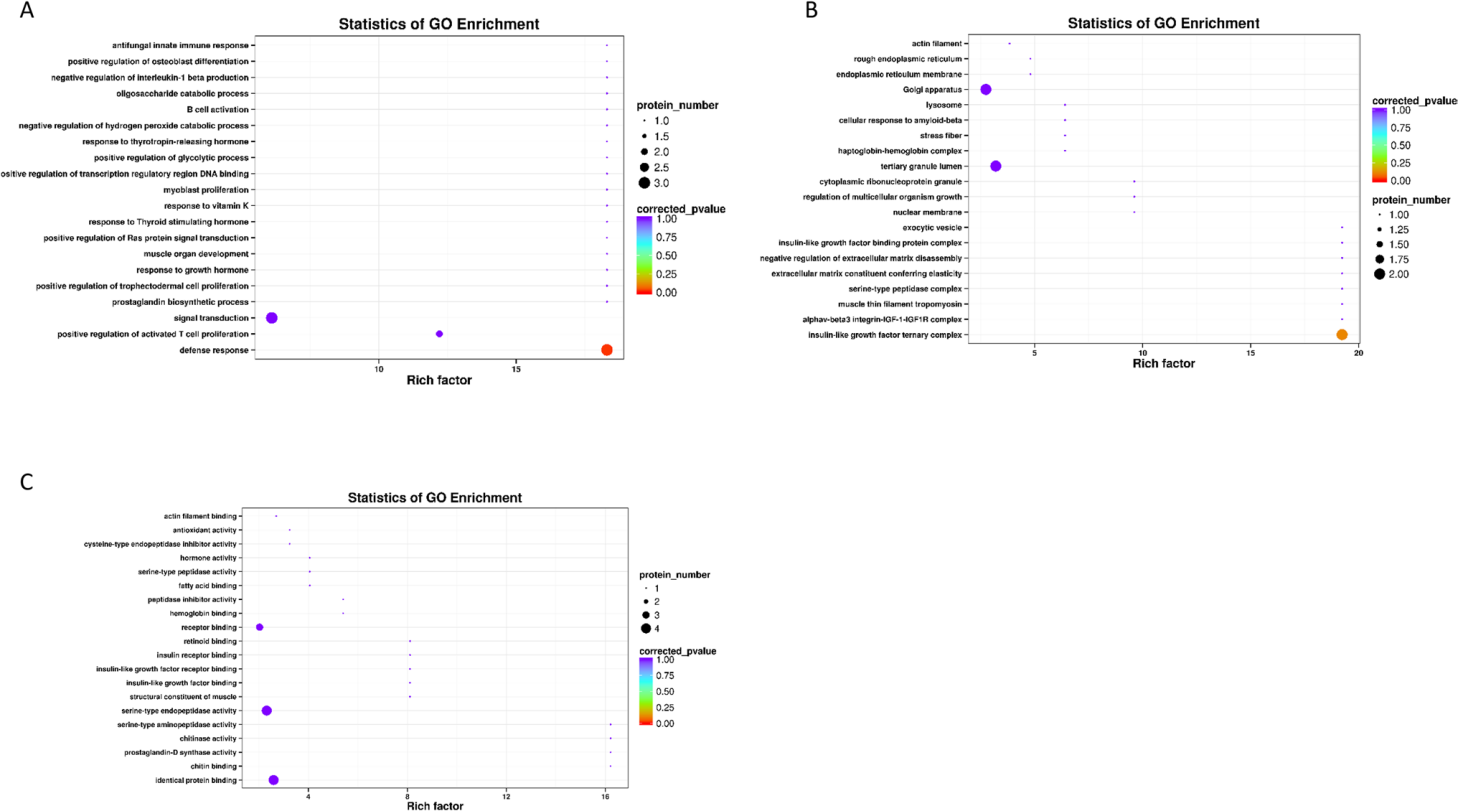
GO enrichment analysis of differential proteins in A vs. B. **A** The top 20 BP terms. **B** The top 20 CC terms. **C** The top 20 MF terms

**Fig. 4:**
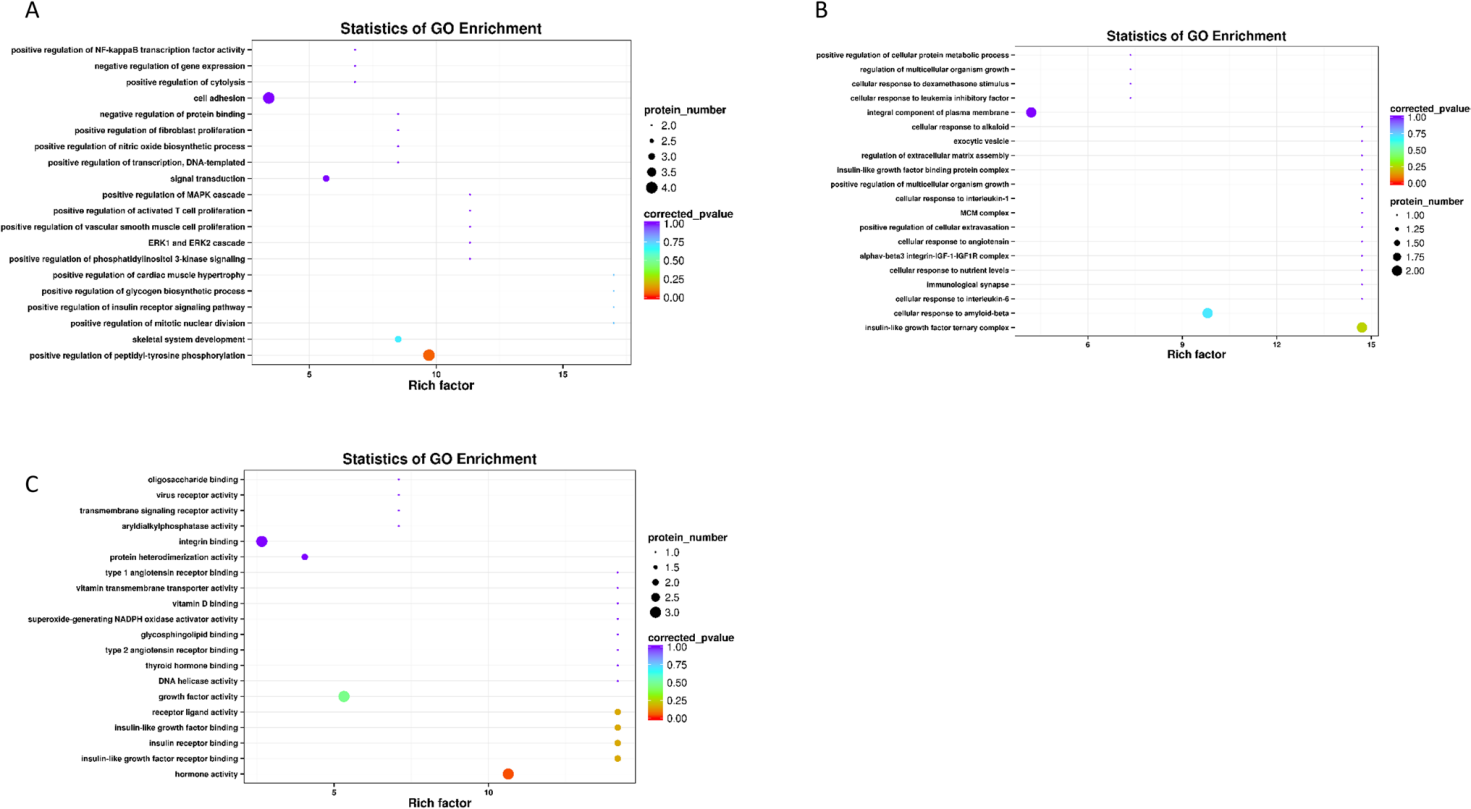
GO enrichment analysis of differential proteins in B vs. C. **A** The top 20 BP terms. **B** The top 20 CC terms. **C** The top 20 MF terms

**Fig. 5:**
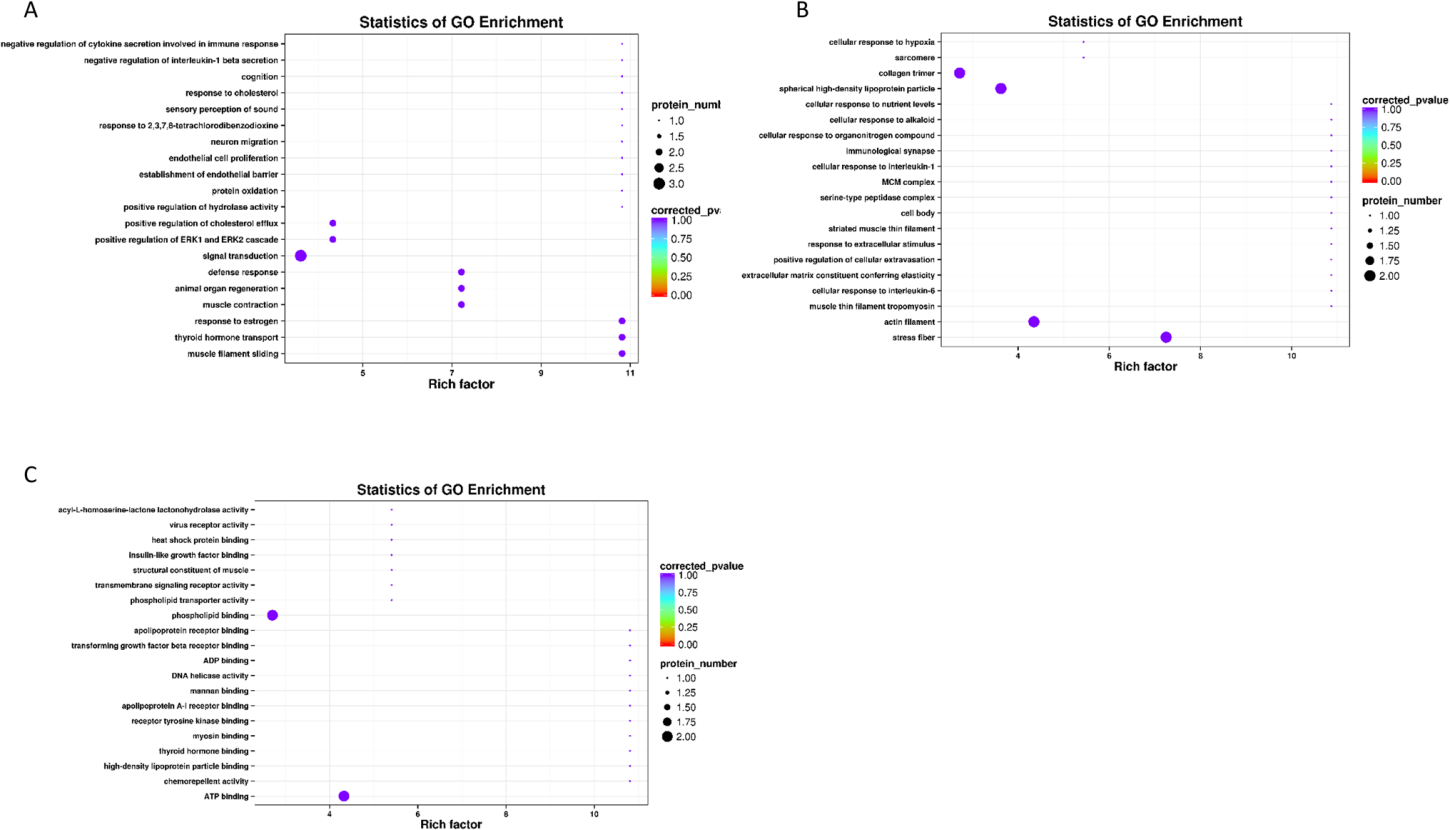
GO enrichment analysis of differential proteins in A vs. C. **A** The top 20 BP terms. **B** The top 20 CC terms. **C** The top 20 MF terms

To further explore the potential functions of differential proteins, we performed GO analyses. GO functional analysis showed that the differentially expressed proteins between groups A and B were mainly related to “insulin receptor binding,” “defense response,” “positive regulation of activated T cell proliferation,” and “cellular response to amyloid-beta” (Fig. 3). In group B vs. C, the differentially expressed proteins were mainly enriched in “positive regulation of activated T cell proliferation,” “insulin-like growth factor binding protein complex,” and “vitamin D binding” (Fig. 4). In group A vs. C, the differentially expressed proteins were mainly enriched in “defense response,” “immunological synapse,” and “insulin-like growth factor binding” (Fig. 5). These results implied that differential proteins are widely involved in immune function and insulin secretion.

### KEGG analysis of differential proteins

**Fig. 6:**
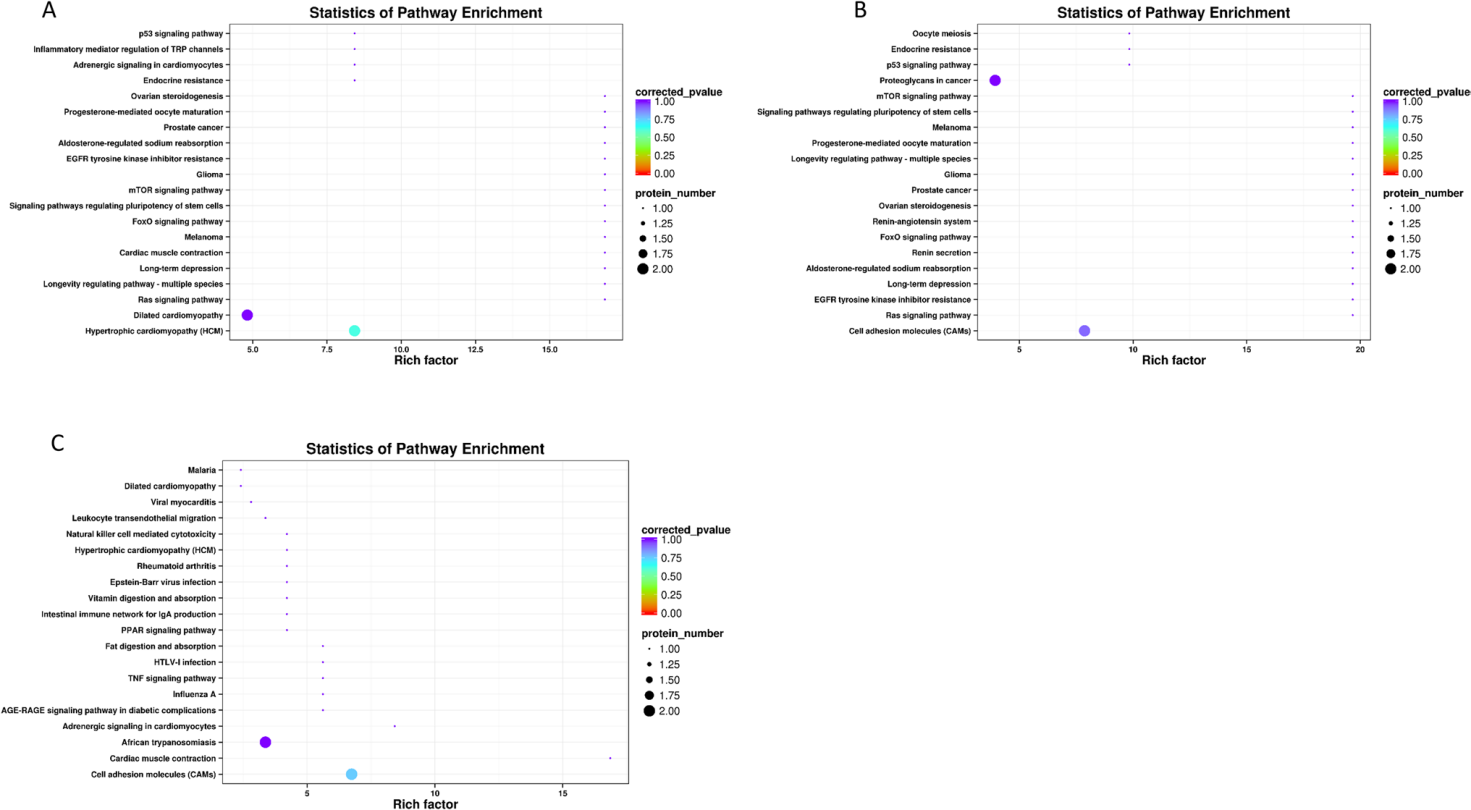
KEGG enrichment analysis of differential proteins. **A** The top 20 KEGG pathways in A vs. B. **B** The top 20 KEGG pathways in B vs. C. **C** The top 20 KEGG pathways in A vs. C

For KEGG prediction, the differentially expressed proteins between groups A and B were mainly involved in “p53 signaling pathway” and “inflammatory mediator regulation of TRP channels” (Fig. 6A). The differentially expressed proteins of group B vs. C and A vs. C were enriched in “cell adhesion molecules” (Fig. 6B, C). In addition, the differentially expressed proteins between A vs. C were related to “vitamin digestion and absorption” and “intestinal immune network for IgA production.” These results indicated that vitamin D deficiency creates an immune regulation disorder.

## Discussion

Vitamin D deficiency contributes to the development of insulin resistance-related diseases, which are generally associated with a compromised immune system [24]. The immune system is divided into innate and adaptive immune systems, and the complement system is an important component of the innate immune system. The imbalance of the complement system leads to the imbalance of the host defense and inflammatory response [25]. Considering the relationship among vitamin D, the immune system, and the complement system, we found that vitamin D deficiency caused glucose metabolism disorders, insulin resistance, and abnormal expression of complement proteins, which induced the abnormal activation of the complement system.

Vitamin D deficiency is common in modern society and is considered an important risk factor for insulin resistance [26]. In the present study, the expression levels of FBG, FINS, and HOMA-IR were upregulated with decreasing vitamin D content. Łagowska et al. reported that vitamin D deficiency can cause insulin resistance [27]. In addition, insulin resistance is often accompanied by hyperglycemia and glucose metabolism disorder [28], which is consistent with the present results. This result may be related to the fact that vitamin D influences insulin sensitivity by regulating extracellular Ca^2+^ concentration and its flux through the cell membrane [29].

Vitamin D deficiency can increase the risk of infection with autoimmune diseases and allergies [30]. We found that a decrease in vitamin D content leads to differentially expressed proteins, and the complement system is the key defense mechanism of the immune system [31]. CFB, a part of the complement substitution pathway, is responsible for C3/C5 convertase activity [32], and its expression is significantly upregulated when the vitamin D content is less than 40 ng/mL in our study, indicating that the complement system is activated and is ready to enter the membrane attack stage [33]. Moreover, the expression of complement component C9 was upregulated with decreasing vitamin D content. C9 is a domain protein that is closely related to immune system diseases [34]. This protein plays a crucial role in the membrane attack complex (MAC) [35], which is a multiprotein complex that can form holes in the membrane of the target pathogen [36]. Furthermore, MAC belongs to the complement system and is the bactericidal weapon of the innate immune system [37]. Continuous upregulation of C9 may represent increased synthesis of MAC, enhanced activation of the complement system, and aggravated immune damage. In the present study, vitamin D deficiency downregulated the expression levels of ICOSL and PI16. According to relevant studies, ICOSL deficiency could cause major defects in immune responses [38]. PI16 is an inhibitor of T cell surface protein peptidase, and its downregulation indicates immune tolerance damage in the body, which may increase the risk of autoimmune diseases [39]. Taken together, the results indicate that vitamin D deficiency can cause the abnormal expression of complement proteins and can then promote the abnormal activation of the complement system, resulting in the suppression of the autoimmune system.

We further conducted a functional enrichment analysis of differential proteins and found that they were mainly involved in cellular functions and pathways related to insulin secretion and inflammation. In addition, the immune system can regulate the endocrine function of islets [40]. The complement system regulates insulin resistance and glucose homeostasis in metabolic diseases [41]. These results further revealed that the abnormally expressed complement proteins caused insulin resistance and glucose metabolism disorder, providing evidence that vitamin D deficiency can cause insulin resistance and blood glucose disorder. However, the underlying mechanisms remain to be elucidated.

In conclusion, the main findings of this study were as follows: (1) vitamin D deficiency caused insulin resistance and glucose metabolism disorder, (2) vitamin D deficiency led to the abnormal expression of complement proteins and the activation of the complement system, and (3) functional enrichment analysis revealed that dysregulated complement proteins were involved in cellular functions related to insulin secretion and glucose metabolism regulation. This study provides a novel reference for further studies on the regulatory effect of vitamin D on the immune system.

## Supplementary Information

Below is the link to the electronic supplementary material.

Supplementary file1 (XLSX 19 KB)

## Data Availability

Data are available via ProteomeXchange with identifier PXD036152.
